# Thermoresponsive Gel-loaded Oxcarbazepine Nanosystems for Nose- To-Brain Delivery: Enhanced Antiepileptic Activity in Rats

**DOI:** 10.1007/s11095-023-03552-7

**Published:** 2023-06-23

**Authors:** Basant A. Abou-Taleb, Samar O. El-Ganainy

**Affiliations:** 1 Department of Pharmaceutics & Pharmaceutical Technology, Faculty of Pharmacy, Pharos University in Alexandria, Alexandria, Egypt; 2 Department of Pharmacy practices, Alexandria University Hospitals, Alexandria University, Alexandria, Egypt; 3 Department of Pharmacology & Therapeutics, Faculty of Pharmacy, Pharos University in Alexandria, Alexandria, Egypt

**Keywords:** anti-epileptic, chitosan nanoparticles, brain delivery, intranasal, oxcarbazepine

## Abstract

**Background:**

Oxcarbazepine (OXC) is a frequently prescribed antiepileptic drug for managing focal and generalized seizures. Its therapeutic benefits are limited by its dose-dependent side effects. Nose-to-brain delivery is a novel route for improving the efficacy of antiepileptics. Drug encapsulation in mucoadhesive nanoparticles offers even more advantages for the nasal route.

**Objective:**

The study aimed to develop oxcarbazepine-loaded chitosan nanoparticles (OXC-NP) added to a mucoadhesive thermo-reversible gel for intranasal delivery and enhancement of antiepileptic activity.

**Methods:**

The formulation was optimized based on entrapment efficiency, polydispersity index, particle size, zeta potential, and *in vitro* release analysis. The therapeutic efficacy of OXC-NP was assessed in an epileptic rat model and compared to intranasal OXC and oral OXC.

**Results:**

The optimized OXC-NPs with chitosan exhibited particle size, zeta potential, and entrapment efficiency of 189 nm, + 31.4 mV ± 2.5 and 97.6% ± 0.14, respectively. The release of OXC was prolonged, reaching 47.1% after 6 h and 55% after 24 h. Enhanced antiepileptic activity of OXC-NP was manifested as decreased seizure score and prolonged survival. Halting of hippocampal TNF-α and IL-6 together with upregulated IL-10 could explain its anti-inflammatory mechanisms.

**Conclusions:**

Intranasal OXC-NP-loaded *in situ* gel represents a promising formulation for enhanced antiepileptic potential achieved at low drug concentrations.

**Supplementary Information:**

The online version contains supplementary material available at 10.1007/s11095-023-03552-7.

## Introduction

Over 50 million people worldwide have epilepsy, which affects all ages, with more individuals in low- to middle-income countries. Besides paroxysmal events, epilepsy might lead to co-morbidities causing neurobiological, psychological, and cognitive burdens [1, 2].

Temporal lobe lesions are the most common type of epileptic seizure in adults. It consists of recurrent focal seizures originating from limbic brain areas such as the hippocampus [2]. Despite the continuous development of pharmaceutical treatments, more than one third of patients do not achieve seizure-free life, primarily because of pharmaco-resistance [3].

Additionally, traditional antiepileptic drugs frequently demonstrate high systemic drug distribution. This usually increases the frequency of unfavorable side effects and drug-drug interactions [4].

Antiepileptic drugs’ effectiveness mostly depends on their sustained brain-based site of action. As a result, throughout the past few decades, various methods have been tried to evade Blood Brain Barrier (BBB) and effectively deliver medications into the brain for therapeutic purposes [5, 6]. Local nasal dysfunctions have traditionally been treated using intranasal medication. Recently, the nasal route has been the best alternative parenteral for brain targeting, as the olfactory area represents a direct association between the brain and the nose [7, 8].

Despite its benefits, nasal administration has some drug penetration limitations due to molecular weight, drug size, and short residence duration in the nasal cavity [9, 10]. To solve these issues, various mucoadhesive *in situ* gelling polymers have been applied in a variety of ways to increase nasal bioavailability and extend the time in which they are in touch with the nasal surface such as temperature dependent systems (e.g. Pluronics and polymethacrylates), pH-triggered systems (e.g. Carbopol, sodium carboxymethyl cellulose and cellulose acetate phthalate) and ion-activated systems (e.g. gellan gum and sodium alginate) [11]. Also small-size mucoadhesive particulate carriers, such as nanoparticles, are employed to target the brain via the nasal route [12]. Chitosan is a frequently used polymer that creates mucoadhesive nanoparticles due to its excellent drug entrapment. Numerous studies have demonstrated how effectively chitosan nanoparticles may improve therapeutic brain targeting [8, 12–21].

Oxcarbazepine (OXC) is a second-generation, highly effective antiepileptic medication authorized as a first-line treatment for focal seizures in children and adults [22]. Oral administration of OXC results in significant systemic distribution to non-targeted organs, which increases the risk of unfavorable dose-dependent peripheral adverse events [4].

A promising method for delivering antiepileptic drugs without modifying the drug’s molecules is nanoparticle (NP)-loaded *in situ* gel. Due to its rapid absorption, biodegradability, acceptability, scale-up practicability, and lack of a burst impact, this system is efficient for delivering therapeutic entities to the brain [23–25]. Using bioadhesive polymers can result in a larger concentration gradient, closer nasal mucosa contact, and increased absorption [26]. *In situ* gels for nasal delivery provide high drug transport, which is typically made practical by the formulation’s prolonged residence period at the absorption site [27]. Suspension of the drug in gel or the drug-to-carrier system can be used to control the release [28, 29]. Different mucoadhesive and bioadhesive polymers have been studied for NP-loaded *in situ* gel for central nervous system (CNS) therapeutic drug delivery via the nasal route, including antidepressant agents [13], anti-Parkinson’s syndrome agents [30], anti-Alzheimer’s agents [31], anti-schizophrenic agents [32] and anti- migraine agents [33].

This research aimed to examine the therapeutic effectiveness of intranasal NPs loaded *in situ* in gels containing OXC for treating epilepsy and assess the pharmaceutical properties and *in vitro* characterization. In addition, *in vivo* efficacy was evaluated by assessing the antiepileptic potential of OXC-NPs loaded *in situ* gel in temporal lobe epilepsy induced via pilocarpine injection in rats. The convulsive behavior of rats treated with OXC-NP was compared to intranasal oxcarbazepine gel and solution. The antiepileptic potential was assessed based on hippocampal neuroinflammatory markers and histopathological changes. This work is the first to investigate intranasal brain targeting of oxcarbazepine chitosan nanoparticles loaded *in situ* gel on both pharmaceutical and therapeutic profiles.

## Materials and Methods

### Drugs and Reagents

Oxcarbazepine (OXC) powder was a gift from Andalus Pharmaceutical-Industry, Cairo, Egypt. Chitosan (CS) (MW equal 200kDa, degree of acetylation more than 90%) was obtained from Alpha Chemika, India. Sodium tripolyphosphate (TPP) was purchase from Loba Chemie, Mumbai-India. Pluronic F-127 was received as free gift sample from Pharco Pharmaceutical Industry, Alexandria, Egypt. Sodium carboxymethylcellulose (SCMC) was purchased from medium viscosity, Loba Chemie, Mumbai-India. Polysorbate 80 (Tween 80) was purchased from El-Nasr Pharmaceutical Chemicals Co. (Qaliubiya, Egypt). Cellulose acetate dialysis bag (VISKING®, Serva Electrophoresis, GmbH, Germany), with a molecular weight cut-off of 12, 000–14,000. All other chemicals and solvents were of analytical reagent grade during the preparation and evaluation of formulation.

### Preparation of OXC-Loaded Chitosan Nanoparticles

Drug-loaded Chitosan NPs (CS NPs) were prepared using tripolyphosphate (TPP) and chitosan ionic gelation [34]. Three chitosan solutions (1, 2, and 3 mg/ml) were dissolved in acetic acid (1% v/v). After the dissolution of chitosan, pH was adjusted to 4.5 using 5 N sodium hydroxide (NaOH). Three different tripolyphosphate (TPP) solutions were dissolved in deionized distilled water at 0.5, 1, and 1.5 mg/ml. Nine formulations were obtained by combining equal volumes (each 10 ml of chitosan solutions combined with 10 ml of TPP solutions) (Table I). Oxcarbazepine (OXC) (50 mg) was dissolved in chitosan solution by dissolving the drug in the least amount of acetone and then added to the chitosan solution. For the creation of the CS-OXC complex, the solutions were stirred on magnetic stirrer at 300 rpm at room temperature for 24 h [15]. After 24 h, 1% Tween 80 was added as a stabilizing agent to prevent aggregation formation. To ensure the creation of a stable colloidal dispersion, the TPP-solution was added to the chitosan-solution while magnetic stirring at 1200 rpm continued for 30 min at room temperature. Three distinct concentrations of chitosan and three different concentrations of TPP solution were combined to create various formulations (Table I). Before being characterized, OXC-loaded CS NPs were maintained at room temperature for 24 h to allow for adequate ionic gelation and cross-linking formation. Each formulation of nine formulations was visually observed and categorized into three categories: opalescent suspension, clear solution, and aggregates. The opalescent suspension corresponded to small particles. After that, the resulting chitosan NP suspension of each formulation was centrifuged for 30 min at 14000 rpm and 4°C.

**Table I:** Oxcarbazepine (OXC) loaded chitosan nanoparticles formulations composition and physicochemical characteristics

Formulation code	CS concentration (mg/ml)	TPP concentration (mg/ml)	Visual observation*	Particle size ± SD (nm)	PDI ±SD	Zeta potential (mV± SD)	%EE ± SD (%)
F1	1	0.5	√	187 ± 2.96	0.280 ± 0.04	+24.0 ± 1.29	95.3 ± 0.25
F2	1	1	x	286 ± 13.97	0.328 ± 0.07	+14.5 ± 1.20	95.7 ± 0.35
F3	1	1.5	x	741 ± 89.64	0.380 ± 0.05	+10.9 ± 0.96	96.7 ± 0.40
F4	2	0.5	√	189 ± 16.70	0.315 ± 0.01	+31.4 ± 2.58	97.5 ± 0.06
F5	2	1	x	589 ± 17.86	0.475 ± 0.05	+16.7 ± 2.08	96.5 ± 0.30
F6	2	1.5	x	1355 ± 9.58	0.716 ± 0.39	+10.9 ± 1.72	97.4 ± 0.15
F7	3	0.5	√	270 ± 23.0	0.353 ± 0.02	+16.5 ± 3.59	95.9 ± 0.10
F8	3	1	x	420 ± 18.3	0.518 ± 0.40	+17.6 ± 1.82	97.5 ± 0.45
F9	3	1.5	x	1262 ± 269	0.644 ± 0.38	+16.7 ± 0.75	96.7 ± 0.35

All formulations contain 50 mg OXC in 10 ml volume (CS solution 1:1 TPP solution), 0.1 mL 0.1% v/v Tween 80 stabilizers.

**CS**, chitosan; **TPP**, tripolyphosphate; **PDI**, polydispersity index; **%EE**, % Entrapment Efficiency. * Opalescent colloidal dispersion (√), Aggregates (x).

### Statistical Design

Using Design Expert^®^ software V13, an experimental design of Box-Behnken was used to determine the effects of various factors on the properties of the prepared chitosan nanoparticles (Stat Ease, Inc., MN, USA) [35]. The concentration of chitosan (mg/ml) (A or X1) and the concentration of TPP (mg/ml) (B or X2) were the two independent variables. Entrapment efficiency (Y3: EE), zeta potential (Y2: ZP), and particle size (Y1: PS) were the observed responses. Table I provides the compositions of the formulas for the generated chitosan NPs. Nine formulas with nine runs were incorporated into this design. All nine formulations were stored at 4°C in refrigerator till characterized. The optimized formula was then chosen to be loaded in thermoresponsive mucoadhesive *in situ* nasal gel based on the predicted desirability values.

### Preparation of Thermoresponsive Mucoadhesive in - situ Gel

In - situ gels based on Pluronic F-127 were made using the cold technique described by Schmolka [36]. The calculated amounts of Pluronic F-127 (18% w/v) and 0.75% w/v of sodium carboxymethyl cellulose (SCMC) (mucoadhesive hydrogels) were used to produce thermoreversible mucoadhesive transparent sols for additional characterization. [27, 33].

The calculated amount of Pluronic F-127 (18% %w/v) was dispersed in cold distilled water at 4°C, in an iced jacket using magnetic stirrer. Dispersions were then stored in a refrigerator overnight to get clear sol [37].

0.75% w/v of SCMC (mucoadhesive hydrogels) was prepared by dissolving SCMC hydrogels in a predetermined quantity of distilled water at room temperature. The gels were subsequently placed in a refrigerator at 4°C. In a thermostatically controlled ice jacket, 18%w/v Pluronic F-127 was then gradually added while being continuously stirred with a magnetic stirrer. After that, dispersions were cooled overnight to produce transparent sols for additional characterization [37].

### Preparation of OXC NP - Loaded in - situ Gel

Following the previous ionic gelation technique, the NPs were added to 18% PF127 and 0.75% SCMC clear homogeneous-solution in which NPs were dispersed [33, 38]. The optimized nasal in-situ gel formula was stored at 4°C in refrigerator till characterized.

### Characterization of an Intra-Nasal Formulation

#### Characterization of OXC-Loaded - Chitosan NPs

Nine formulations for several factors, including entrapment efficiency, particle-size, zeta -potential, size -distribution, and morphology, were identified.

Zeta-potential (ZS), distribution (poly dispersity index (PDI)), and particle size (PS) of nine formulations were characterized using Zetasizer-Nano ZS (Malvern, UK) [13]. The means and standard-deviations (SD) were calculated after each measurement was carried out in triplicate.

##### Drug Entrapment Efficiency (EE)

By centrifuging the formulation at 14000 rpm for 30 min at 4°C, the amount of drug entrapped in NPs was measured. The NPs -settled down, and a free drug was present in the supernatant. The UV-1800 Shimadzu spectrophotometer (Japan) was used to evaluate the supernatant at λ _max_ = 321 nm [39]. The study was carried out in triplicate, with the given mean values.

Entrapment efficiency (EE%) of the NPs was determined per the following equations:

1 $$\frac{\textbf{EE}\%=\textbf{Total}\ \textbf{drug}\ \textbf{added}-\textbf{Free}\ \textbf{drug}\ \textbf{in}\ \textbf{supernatant}\times \textbf{100}}{\textbf{Total}\ \textbf{drug}\ \textbf{added}}$$

Where the total drug represents the whole concentration of drug added to the system, and the free drug represents the concentration of free drug within the supernatant.

##### Morphology

The drug’s entrapment inside chitosan NPS was visualized using transmission electron microscopy (TEM), and the produced OXC-CS NP’s size and inside morphology was analyzed using (TEM, JEOL JEM-1400 PLUS, Joel Ltd., Tokyo, Japan). The outside morphology and size of the prepared OXC- CS NP were examined using Scanning electron microscopy (SEM) (SEM, JEOL JSM-IT 200, Joel Ltd., Tokyo, Japan). TEM and SEM sample preparations are found in details in (Supplementary Methods).

##### Drug Content

10 mg of freeze - dried OXC-NPs were dissolved into a 50 ml mixture solution (1% acetic acid: acetone: Distilled water) at a ratio of 1: 65: 34 and kept overnight [8, 40, 41]. The soaked solution was filtered by a 0.45 micropore filter & analyzed in a UV spectrophotometer at λ max= 321 nm [39].

#### Characterization of Thermoreversible OXC NP-loaded *in situ* gel

##### pH

Formulations’ pH was monitored using sensitive micro-processor pH meter, calibrated at room temperature using standard buffers of pH7 [42].

##### Gelling Temperature

The temperature at which liquid phase transforms into the gel is determined as the gelling temperature. To carry out the study, gelling temperature of OXC NP-loaded *in situ* gel & placebo *in situ* gel were determined by thermometer in water bath on the basis of fluidity of the developed formulation. Two millilitres of the test formulation were added to a 10 mL volume, 1 cm diameter test tube, which was submerged in a water bath heated to the required temperatures (24°C-35°C) and allowed to acclimate for 5 minutes at each new setting. The samples were tested for gelation, which was defined as occurring over 30 seconds by the flow or no-flow criterion, or when the meniscus ceased to move upon tilting through a 90° angle [43, 44].

##### Gelling Time

The test tube inverting approach was used to establish the temperature range under investigation [43]. At the determined gelling temperature, two millilitres of the formulation were maintained in a water bath. Every several seconds, the tube was removed and inverted to check on the sample's condition.

*In situ* gels are characterized by their capability to gel at a particular temperature. To establish the gelling time, the colloidal solution was kept at its gelation temperature in water bath. Then the time essential for the conversion to take place was observed and this corresponds to the gelling temperature.

##### Gel Strength

Gel strength was determined according to modified Choi *et al* method [45]. It was measured by adding 35 g of the formulation to a 100 cm^3^ graduated cylinder measuring and gelling it at a thermostat-controlled at 37°C. Piston (20-g ) was carefully put onto the gelled fluid and allowed to freely pierce 3 cm^3^ of the gel. The time needed for a certain weight to sink 3 cm3 in the gel was determined, which is directly correlated to gel strength.

##### *In-vitro* Mucoadhesion

The displacement method of Nakamura, F., *et al*. [46] was used to conduct *in-vitro* mucoadhesion testing. The specified gelled mass was positioned at a 60° angle on top of agar 1%/mucin 2% w/v cast on a glass plate in an incubator set at 37°C [47]. Up to ten hours, the displacement (the gel mass moving lower) was recorded hourly in centimetres (cm). Thus, the displacement of the gel has an adverse relationship with the adhesion potential. The average values for each measurement were determined (±SD) in triplicates.

##### Rheological Studies (Viscosity)

The rheological behaviour of sols for both drug loaded gel and NP-loaded gel was evaluated using rotating viscometer (Brookfield DV II-RV, USA) coupled with S-15 spindle at 100, 200 rpm. To evaluate the viscosities of the formulations, temperatures were kept at 24 ± 1°C and 34 ± 1°C (room temperature and nasal temperature respectively) [27, 37].

##### *In - vitro* OXC-Release Study

The dialysis-bag diffusion technique was used to assess the *in- vitro* release of OXC [48]. The *in vitro* release rates of OXC suspended in water, OXC-loaded gel and OXC-NP-loaded gel from the optimized chitosan nanoparticle formulation (F4) distributed in the mucoadhesive *in- situ* gel was carried out in a dissolution medium consisting of simulated –nasal - fluid (SNF) at pH 6.8 [33, 49, 50] and ethanol in a ratio of 80:20 to fulfill sink conditions [51, 52]. The test formula contained 5 mg of OXC and was placed within a pre -moistened cellulose-acetate dialysis-bag and sealed at both ends.

The receptor compartment (glass beaker) contained 50 ml of dissolution medium(20% ethanol and 80% SNF), in which the dialysis bag was submerged [53]. The temperature was maintained at 34 ± 0.5°C while it was shaken at 50 rpm in a water-bath horizontal-shaker with a thermostat, simulating nasal conditions. At predefined intervals (1, 2, 4, 6, 24, and 28 h), a 2 ml sample of the receiver medium was withdrawn and replaced with 2 ml volume of fresh-medium to maintain sink-condition. At λmax of 321 nm, the samples were spectrophotometrically examined for drug-content. The order of drug release from the various formulations was determined using DD-solver software, fitted to four different kinetic models (zero-order, first-order, Higuchi-diffusion model, or Peppas-Korsmeyer), and used to characterize the release kinetics and, consequently, the drug-release mechanism [54]. The data were analyzed using linear regression equations per the constructed calibration curve of OXC in a dissolution medium (at a concentration range of 10–100 μg/ml . All measurements were made in triplicates.

### *In vivo* Pharmacodynamics Studies

#### Experimental Animals

Male Sprague-Dawley rats weighing 210–230 g were obtained and housed in the animal house, Faculty of Pharmacy, Pharos University in Alexandria. They were kept under observation for at least one week before the study, with food and water at free access. All procedures were performed per the Research Ethical Committee of the Faculty of Pharmacy, Pharos University in Alexandria, and complied with the ARRIVE guidelines and the National Research Council’s Guide for the Care and Use of Laboratory Animals. The ethics committee reviewed and approved the study protocol, with approval No: 01202109263031.

#### Induction of Seizures and Experimental Protocol

Intraperitoneal pilocarpine injection (380 mg/kg) was used to induce seizures in rats. Pilocarpine, in high doses, is known for its ability to induce temporal lobe epilepsy in animals, status epilepticus, and brain damage. Rats were randomly distributed into 5 groups (n = 6): **NOR**: denotes normal rats **PLC** : denotes pilocarpine treated rats representing positive control rats, **OXC SUS** : denotes rats treated with oral Oxcarbazepine suspension following pilocarpine injection , **OXC GEL :** denotes rats treated with intranasal Oxcarbazepine gel following pilocarpine injection, **OXC- NP:** denotes rats treated with intranasal Oxcarbazepine-chitosan nanoparticles in gel. The dosage of oxcarbazepine was 0.8 mg/kg based on a pilot study and formerly reported work [55]. All oxcarbazepine preparations were administered 1 hour before pilocarpine injection. OXC SUS was administered orally via oral gavage, while OXC GEL and OXC-NP were administered intranasally using micropipette. Intranasal solutions were administered in one nostril using micropipette while held in upright position. After intranasal application, rats were held in an upright position for 10 sec to allow the flow of solution with normal inhalation. Detailed experimental groups can be found in Table II.

**Table II:** Experimental protocol for *in vivo* study

Group	Treatment
NOR	Untreated group
PLC	treated with intraperitoneal pilocarpine (100 μg/μl)
OXC SUS	treated with oral Oxcarbazepine suspension (0.5 μg/μl) ^¥^
OXC GEL	treated with intranasal Oxcarbazepine gel (5 μg/μl) ^¥^
OXC- NP	treated with intranasal Oxcarbazepine-chitosan nanoparticles in gel (5 μg/μl) ^¥^

¥ all oxcarbazepine preparations were administered 1 hour before pilocarpine injection. Pilocarpine dose = 380 mg/kg, while oxcarbazepine preparations dose = 0.8 mg/kg. Intranasal solutions were administered in one nostril using micropipette while held in upright position.

#### Evaluation of Convulsive Behavior

Following treatment administration, rats were placed in a 30 cm x 40 cm cage for seizure observation for up to 90 min. The following data were recorded: latency to the first seizure, seizure score, and percentage of rats showing status epilepticus [55, 56]. The convulsive behavior was scored to correspond to Modified Racine’s scale [57]. Each group was distributed into two halves (n = 6). The first half was left to record the 24 h survival. The other animals were sacrificed, and hippocampi were dissected and frozen at -80°C for additional biochemical study. For histological analysis, half of the brains were placed in formalin.

#### Biochemical Determination

Isolated hippocampi were homogenized, and aliquots were used for the following determinations using the ELISA technique: IL-10, TNF-α, and IL-1β, levels (MyBioSource, USA). Each sample’s protein content was measured using a colorimetric technique, and all parameters were represented as pg/mg protein.

#### Histopathological Examination

The isolated brain halves were removed, cleaned with cold phosphate-buffered saline, and then fixed in 10% formalin. Blocks with paraffin were made and sectioned. Hematoxylin and eosin (H&E) staining was applied to sections before light microscopy analysis. The brain was evaluated for any histological alterations under a light microscope, with emphasis on the hippocampus.

### Correlations Investigated

Plotting the mean % release after 6 h *versus* latency to the first seizure, the average seizure score, rats showing status epilepticus, and 24 h survival (%) was used to investigate the quantitative correlation between *in vitro pharmaceutical dissolution data with in vivo* pharmacodynamic data. To assess the correlation’s robustness and determine its strength and significance, a linear-regression analysis was used. R^2^ & p-value were determined [58, 59].

### Statistical Analysis

The findings of each *in vitro* test were provided as the mean ± SD after being performed three times. One-way-variance analysis was used to evaluate the data statistically. Individual differences were evaluated using a nonparametric post-hoc test. A difference with a p-value of < 0.05 was used to determine statistical significance. The collected *in vivo* results were provided as mean ± S.E.M. (n = 6). A one-way analysis of variance (ANOVA) followed by Newman-Keuls-multiple comparison test were used to analyze the results. GraphPad-Prism software was used for all statistical analysis V5.0.

## Results and Discussion

### Effect of Chitosan and TPP Concentrations on P. S, PDI, zeta potential and % EE of OXC-CS-NPs

Chitosan and TPP concentrations significantly affected the prepared OXC-CS NPS particle size. The formulation was optimized by investigating the effects of three levels of TPP concentration (0.5, 1, and 1.5 mg/ml) and three levels of chitosan concentration (1, 2, and 3 mg/ml) on the formed NPs’ zeta-potential, entrapment-efficiency (%EE), poly dispersity index (PDI), and particle-size (PS).

The development and optimization of nasal medication delivery systems depended significantly on PS. A study showed that smaller particles could penetrate the mucosal membranes more deeply [35]. Therefore, creating formulas with little PS was one of the study’s primary objectives.

The polynomial quadratic model was fitted to the PS values. The difference between the predicted R^2^ (0.5851) and the adjusted R^2^ (0.7590) was acceptable (< 0.2). The employed quadratic model was successful in detecting a response value because the value of adequate precision was >4 (7.84) [60]. The results of PS were analyzed using the following equation:

2 $$\textbf{Particle}\ \textbf{size}\ \left(\textbf{PS}\right)=+588.78+123.00\ast \mathrm{A}+452.00\ast \mathrm{B}+109.50\ast \mathrm{A}\mathrm{B}$$

**Fig. 1:**
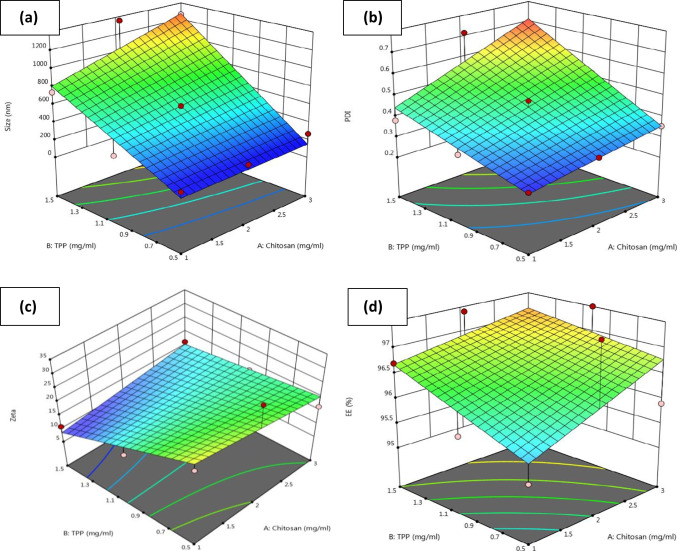
3D response surface plots for the effects of Chitosan (A) concentration and TPP (B) concentrations; [(**a**) on particle size], [ (**b**) on PDI], [ (**c**) on ZP] and [(**d**) on EE] of OXC- CS NPs.

As illustrated in Fig. 1a, the incorporation of different CS concentrations (A) (mg/ml) and concentrations of TPP (B) (mg/ml) demonstrated a p-value of 0.0169 with statistically significant effects on the mean PS. The PS of the produced formulas was primarily dependent on TPP concentration, according to statistical analysis. Moreover, the results shown in Table I showed that upon increasing the TPP concentration from 0.5 to 1.5 mg/ml, a highly significant increase in particle size percentage occurred (p = 0.004).

Furthermore, the estimated PDI values are shown in Fig. 1b and Table I. Except for the F5, F6, F8, and F9 formulas, all of the OXC-CS NPs formulations demonstrated PDI values in the 0.28–0.716 range, which is an acceptable midrange [61]. The incorporation of different CS concentrations (A) (mg/ml) and TPP concentrations (B) (mg/ml) showed a significant effect on the mean PDI (p = 0.0188). PDI values were statistically evaluated utilizing polynomial quadratic analysis (Fig. 1b). The value of adequate precision was > 4 (8.54). The PDI values were analyzed using the following equation:

3 $$\textbf{Polydispersity}\ \textbf{Index}\ \left(\textbf{PDI}\right)=+0.4454++0.0878\ast \mathrm{A}++0.1320\ast \mathrm{B}++0.0478\ast \mathrm{A}\mathrm{B}$$

In contrast, the values of ZP listed in Table 1 ranged between +10.9 and + 31.4 mV. The generated nanodispersion could not aggregate due to the significant repulsion forces provided by the high ZP [35]. The incorporation of different CS concentrations (A) (mg/ml) and TPP concentrations (B) (mg/ml) showed no significant effect on the mean ZP (p = 0.0937). Additionally, the incorporation of different CS concentrations (A) (mg/ml) factors demonstrated an insignificant effect on the ZP values (p = 0.0812). In contrast, the incorporation of different TPP concentrations (B) (mg/ml) significantly impacted the values of ZP, with p-values equal to 0.0288, as shown in Fig. 1c.

All nano gel formulas had EE percentages above 95% (Table I). These findings demonstrated the compositions’ potential for successfully entrapping the drug inside the CS nanoparticles. The incorporation of different CS concentrations (A) (mg/ml) and TPP concentrations (B) (mg/ml) showed an insignificant effect on the mean EE (= 0.5223). P-values of the factors mentioned above equaled 0.2938 and 0.3519 for chitosan (A) and TPP (B) different concentrations, respectively (Fig. 1d).

### Choosing the Optimized OXC NP Formulas

The ionic gelation method was used to create OXC-loaded chitosan NPs successfully. To choose the optimized OX-CS NP formulas with the lowest PS, highest EE, highest ZP, and most appropriate PDI, the values of desirability were assessed. In the case of optimized NP formulae, the greatest desirability value was 0.999 (Supplementary Figure 1). The optimized nasal formula was the F4 formula, which contained chitosan (2 mg/ml) and TPP (0.5 mg/ml) with an equal volume ratio. The F4 formula was selected to be incorporated into a thermoreversible gel for nasal application. The polydispersity index (PDI) of the optimized F4 chitosan NPs was 0.315 ± 0.01, the average particle size was 189 ± 16.70 nm, and the zeta potential was +31.4 ± 2.58. The EE% for F4 was found to be 97.5 ± 0.06% (Table I). The optimized OXC NP formula showed good stability during 3 months storage at 4°C. This may be due to the use of 1% Tween 80 as stabilizing agent which allowed the production of small chitosan nanoparticles and kept the PS at suitable values for nasal application up to 3 months at 4°C.

### Morphology and other Parameters

**Fig. 2:**
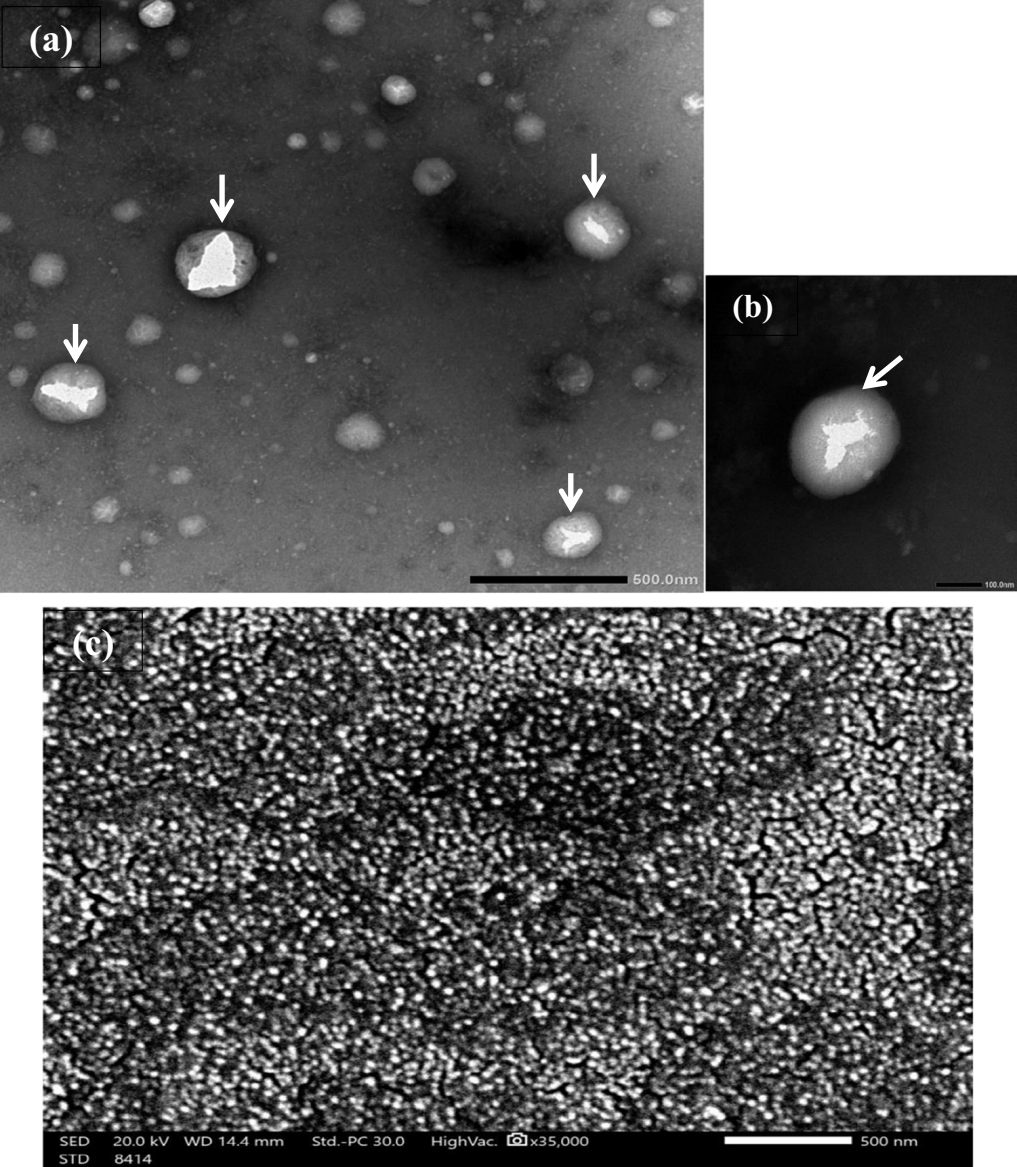
TEM photographs of (**a**); OXC-loaded Cs-NP nanoparticles and (**b**); a magnified single Cs-NP nanoparticle (the arrows point at the drug encapsulated within the nanoparticles) and (**c**); SEM photographs of OXC-loaded Chitosan nanoparticles.

The OXC-CS NPs’ TEM and SEM images (Fig. 2) showed that the NPs are generated at a small size and spherical-shape. Fig. 2 shows the entrapment of the drug inside chitosan nanoparticles (CS NP).

Freeze-dried nanoparticle product was evaluated for product yield and drug content. The drug content for the optimized F4 formula was found to be 97.45%, nearly equal to the % entrapment efficiency of the same formula. These results prove the high stability of the (F4) nanoparticle formula chosen for OXC loaded in thermoresponsive in-situ gel.

### Preparation & Characterization of OXC-CS NP -Loaded *in- situ* Gel

The improved CS-NP formula (F4) was combined into an *in- situ* gel with mucoadhesive properties to confirm straightforward intra nasal delivery, prolong its staying time, and enhance absorption. Pluronic F-127 concentration used for thermoresponsive *in- situ* gel formation was 18% w/v, where the gel phase was generated when the concentration was > the micellar concentration [62]. According to a previous report, a temperature range of 25–37°C is ideal for nasal application [63]. For this reason, the product’s gelling temperature should be greater than 25°C to prevent challenges with formulation manufacturing, handling, and administration [64]. Since the nasal cavity is around 34°C in temperature [63], our objective was to develop a F-127-based thermoreversible gel that changes to a gel state between 25 and 34°C and a liquid state below 25°C, causing early loss of the delivered medications due to nasal-clearance [27]. According to a previous study, the sol- to -gel transition temperature is reduced as the Pluronic F-127 concentration increases [65]. With the addition of various mucoadhesive polymers, such as low-molecular-weight sodium carboxymethyl cellulose (SCMC) at 0.75% concentration, the formulation’s nasal cavity residence time could be prolonged. In this experiment, Pluronic F-127 was fixed at an 18% concentration to achieve the proper gel transition-temperature in the nasal-cavity. Furthermore, the impact of SCMC inclusion on the transition temperature of the Pluronic F-127 gel was assessed. According to the characterization data, the thermoresponsive *in- situ* gels made from the (18% w/v F-127) and (0.75% w/v SCMC) mucoadhesive polymers performed satisfactorily concerning *in- vitro* (mucoadhesion, gelling-time & temperature, gel-strength, pH, and viscosity) (Table III).

**Table III:** Composition and various physical parameters characterized for the *in situ* gel and for both OXC loaded *in situ* gel and optimized NP-loaded *in situ* gel

Formula name	CS conc. (mg/ml)	TPP conc. (mg/ml)	CS: TPP	F-127 (%)	SCMC (%)	Gelling temperature (°C)	Gelling time (Sec.)	pH ± SD	Viscosity at 25°C (cP ± SD)		Viscosity at 30°C (cP ± SD)		Gel strength (Sec. ± SD)	*In-vitro* mucoadhesion
									100 rpm	200 rpm	100 rpm	200 rpm		
Gel	-	-	-	18	0.75	26.5	5	7	-	-	-	-	39	Good
OXC Gel	-	-	-	18	0.75	26.5 - 27	5	6.8	-	-	-	-	40	Good
OXC-NP Gel	2	0.5	1:1	18	0.75	29	3	6.4	4213±153	2128±165	4917±119	2481±9.80	42	Good

Mucoadhesion testing revealed that when positioned over the agar/mucin plate, optimized formula F4 NP Gel (OXC NP Gel), OXC Gel, and placebo gel did not move for up to 12 h, indicating acceptable adhesion and being rated as "Good" acceptable mucoadhesion (Table III).

The optimal formula of the OXC NP gel was at 29°C, which was appropriate and within the nose temperature (Table III). The optimized formula would, therefore, be liquid at room-temperature and change to a gel after being administered into the nostril. The increased contact time in the nasal-cavity could be referred to the thermal reversibility of F-127/SCMC gel.

The loaded OXC-NP in gel showed rapid gelation time (Table III) at the cavity of the nose, which is significant concerning nasal *in- situ* mucoadhesive gels to prevent the formulation’s rapid clearance in the solution state and to guarantee that the medicine is retained on the nasal mucosa for a considerable time [66].

The viscosity of the formulation of the thermosensitive *in- situ* nasal mucoadhesive gel ought to be ideal for straightforward instillation, and after that, it is transformed into the gel. 35°C, which represents the nasal body temperature, and 25°C, which represents room temperature, were utilized to estimate the viscosity (Table III). Because of their thermosensitivity, the formulations were liquid and had low viscosities at 25°C; as the temperature rose, they began to gel and exhibit greater viscosities. The viscosity of OXC NP gel at 25°C showed 4213 and 2128 values at 100 and 200 rpm, respectively; however, at 35°C, it was substantially higher with values equal to 4917 and 2481 at 100 and 200 rpm, respectively. The following could be explained regarding the behavior of temperature-dependent gelation: Pluronic F-127 chains have prolonged coils covered in a hydration layer at 25°C. However, the H-bond between PPO units and water dissolves at higher temperatures, causing desolvation. As the polymer chains go closer, they interact more, which raises viscosity [67]. It should be mentioned that the mucoadhesive polymer (SCMC) utilized and the Pluronic F-127 content contributed to the gels’ viscosity. The attraction of the oxygen-atom of the Pluronic F-127 ether to the protons of water is thought to be the catalyst for hydrogen-bonding in aqueous-systems, which resulted in the formation of the block copolymer F-127 thermosensitive gels. The addition of hydroxyl group-containing compounds, such as SCMC, is anticipated to increase hydrogen bonds, increasing the viscosity of the developed formulations [68].

To ensure that the *in situ* gel formulations are suitable for intranasal instillation, the pH of all formulations had to be established. Although the mucosa’s of the nose normal physiological pH is between 4.5 and 6.5, it could tolerate formulations with a pH range of 3 to 10 [69]. The *in- situ* gel formulations’ pH values fell between 6.4 and 7 (Table III). As a result, it was expected that no formulations would have any negative consequences related to pH.

Because gel-strength times greater than 50 seconds were too stiff and might irritate or harm the mucosal surfaces, while values < 25 s would not be capable of maintaining their integrity and could erode rapidly, *in situ* gel formula values fell between 39 and 42 s (Table III), which was suitable for intranasal instillation [70]. The rise *in- situ* gel-strength with the addition of the mucoadhesive-polymers could be attributed to the formation of hydrogen-bonds between Pluronic F-127 and the mucoadhesive-polymers used in the formulation [33, 62].

### *In- vitro* Drug Release Rates for Optimized OXC- loaded NPS Formula

**Fig. 3:**
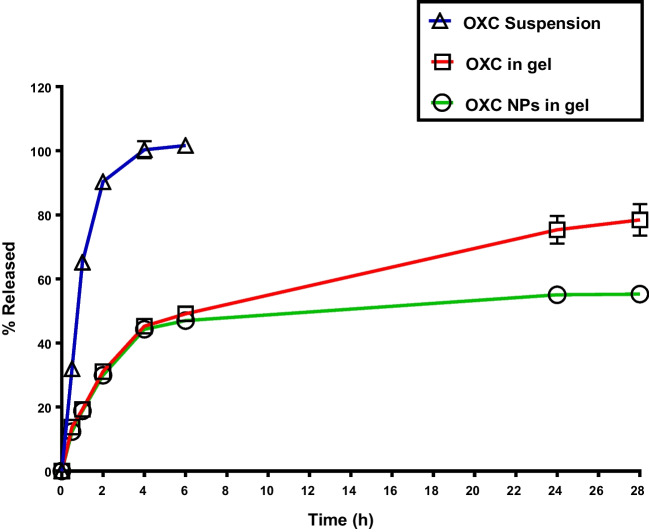
Cumulative release profiles at 34°C of OXC from OXC (suspension), OXC in gel and OXC NPs in gel.

Using the dialysis-bag method, the *in- vitro* cumulative drug-release profiles of OXC suspension, OXC-loaded-gel, and OXC NP-loaded gel were assessed as shown in Fig. 3. The 8:2 ratio of SNF to ethanol was used as the release medium. The solubility of OXC in the release medium was assessed and found to be 280 μg/ml, allowing for the maintenance of the sink-condition. Ethanol was added to dissolution medium because it is one of the methods used for maintaining dissolution sink conditions for a poorly soluble drug [52]. An increase in drug solubilization can be achieved through the addition of organic co-solvents such as ethanol to the dissolution medium [51, 52, 71, 72]. The *in vitro* release profile of OXC suspension showed 100.3% ± 2.6 release at 4 h, while the release profile of OXC in gel and from CS-NP in gel showed (45.24% ± 1.66) and (44.26%± 1.58) respectively after 4 h, then an extended slow release that just reached 55.25 ± 1.02 release % after 28 h for OXC NP-loaded gel. As a result, it could be concluded that both the drug- loaded *in- situ* gel and drug- loaded CS NPs loaded *in- situ* gel demonstrate a biphasic release pattern, with OXC releasing rather rapidly during the early phase (the first four hours) and then more slowly and steadily throughout the following 28 h. OXC drug release was delayed when it was added to F-127-based thermoreversible *in situ* gels with mucoadhesive SCMC polymer compared to OXC oral suspension. According to reports, Pluronic F-127 slowed the drug release rate by reducing the micellar structure’s size and number of water channels. The narrower inter-micelle distance induced more cross-links between adjacent micelles, increasing viscosity and delaying the release of drugs [73, 74]. Diffusion via the gel matrix might release the drug after incorporation. Drug release was extended in OXC-loaded gel and OXC NP-loaded gel formulations, with values of 78.4% in the case of OXC gel and 55.25% for OXC NP-loaded gel, respectively, at 28 h. Usually, the rapid drug release of OXC-NPs gel at the beginning is due to those OXC molecules that are not trapped (free), and those OXC molecules that are entrapped close to the nanoparticle surface diffuse more rapidly and easily [15, 75].

As a result, the tested formulas’ drug release could be organized ascendingly as follows: OXC NP-loaded gel, OXC in gel, and OXC suspension. In this instance, the presence of a gel matrix and the drug’s solubility impacted OXC release.

The hydrogen-bonding between OXC and chitosan polymer, which needed time to break down and allow the medication to diffuse outside the nanoparticles, might be the reason for the significantly slower release of OXC NPs gel than OXC gel that followed the rapid release, in addition to OXC being present in a polymeric matrix [76]. Since the nanoparticles would not demonstrate a premature drug-release before reaching their intended target, the brain, the delayed release is anticipated to be effective *in vivo*. This release pattern is appropriate for the anticonvulsant drug OXC, which requires a prolonged release pattern to maintain the loading dose after an initial rapid release to manage epilepsy [77, 78].

**Fig. 4:**
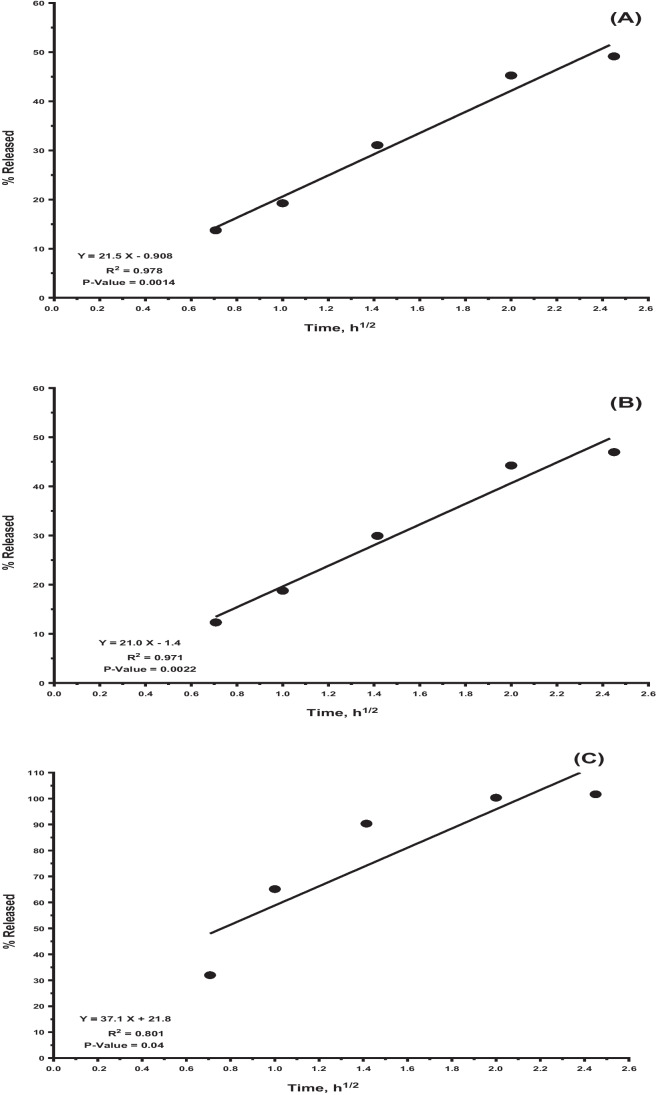
Higuchi release plots; (**a**) OXC gel, (**b**) OXC NP gel and (**c**) OXC Suspension.

Studying drug-release mechanisms & kinetics is essential to accurately describe the drug release or diffusion profile of a delivery-method. The drug release data of the OXC NPs gel formulation were fitted to various models, including the Higuchi-equation, zero-order, Korsmeyer-Peppas, and first-order [79, 80]. Mathematical data modeling revealed that the release pattern of both *in- situ* gels (OXC gel and OXC NPs gel) follow Higuchi order kinetics. In contrast, OXC suspension follows first-order release kinetics (Fig. 4).

OXC release from NPs in gel followed Higuchi model of release kinetics with high (r2) value of 0.971 hence shows that release of drug occurred through diffusion mechanism. The information was fitted into Korsmeyer et al's equation to verify the diffusion mechanism. The diffusional release exponent "n" value was 0.266, while the coefficient r2 value was 0.9283. Values of n < 0.5 support the Fickian diffusion-controlled mechanism for OXC release from the tested in-situ gel OXC CS NPs [79, 80]. The prolongation of the drug release rates could be attributed to the increased viscosity imparted by the developed *in situ* Pluronic F-127 based gels[68]. Additionally, the SCMC employed had a retarding impact since it increased the product’s overall viscosity and distorted or squeezed the Pluronic F-127 micelles’ extra-micellar aqueous channels, where the OXC diffuses. Therefore, the *in situ* gel provides an additional barrier to the release of OXC rather than the chitosan nanoparticles themselves.

### Effect of Oxcarbazepine Preparations on Convulsive Behavior in Temporal Lobe Epileptic Rats

The most frequent type of partial complex seizures in adults is temporal lobe epilepsy, requiring attempting treatment [81]. Following pilocarpine injection, rats developed seizures with an average of 2.64 ± 0.45 seconds. Seizures developed into generalized convulsions, as evaluated by a modified Racine’s scale, and progressed into status epilepticus (continuous convulsions) in 80% of rats (Table IV, Supplementary Figure 2). Treatment with OXC SUS showed the same seizure latency, score, and status epilepticus percent observed in the pilocarpine-treated group. This could be explained by the low dose administered compared to previous epileptic models [82, 83]. Treatment with OXC GEL slightly prolonged the time to the first seizure and induced a lower seizure score, but this was insignificant compared to the PLC group. However, the percentage of rats developing status epilepticus was prominently decreased following OXC GEL treatment. In the OXC-NP group, latency to the first convulsion was significantly higher compared to the PLC, OXC SUS, and OXC GEL groups (Table IV). In addition, the seizure score was significantly lower compared to that recorded in PLC or OXC SUS-treated rats (p < 0.05). Rats showing status epilepticus seizures had prominently declined (27%), similar to that observed with OXC GEL treatment. The percentage of rats surviving for 24 h after pilocarpine injection was higher in both OXC GEL and OXC-NP (2.5-fold) compared with the 24 h survival of the PLC or OXC SUS groups. Intranasal formulations of OXC had previously shown high therapeutic efficacy in animal models of epilepsy [55, 84]. However, this is the first study to show the superiority of these novel formulations in pilocarpine-induced temporal lobe epilepsy, with higher potential noticed with OXC-NP loaded in the gel.

**Table IV:** Effect of oxcarbazepine suspension, oxcarbazepine gel and oxcarbazepine chitosan nanoparticles in gel on pilocarpine-induced seizures and lethality in adult rats

Treatment	Latency to first seizure (sec ± SEM)	Average seizure score (score /group ±SEM)	Status epilepticus (%)	24-hours survival (%)
PLC	2.64 ± 0.45	5.6 ± 0.33	80	20
OXC SUS	2.60 ± 0.60	5.9 ± 0.10	80	17
OXC GEL	3.80 ± 0.66	4.7 ± 0.47	30	50
OXC NP GEL	5.6 ± 0.73^#$@^	4.1 ± 0.45^#$^	27	50

PLC: group treated with pilocarpine; OXC SUS: group treated with oxcarbazepine suspension XC GEL; group treated with oxcarbazepine in gel; OXC NP; group treated with oxcarbazepine chitosan nanoparticles in gel. Statistical analysis was done using one-way ANOVA followed by Student-Newman-Keuls multiple comparison test. # p<0.05 vs PLC, $ p<0.05 vs OXC SUS, @ p<0.05 vs OXC GEL

### Effect of Oxcarbazepine Preparations on Hippocampal Neuroinflammatory Markers

The pathophysiology of epilepsy is heavily influenced by neuroinflammation. The release of inflammatory mediators precedes epileptic seizures and potentiates their generation. They contribute to astrocytes, microglia activation, and neuronal cell damage, facilitating the transition to brain pathology [85]. Such a process is thought to be stimulated by the decline of anti-inflammatory molecules. During seizures, inflammatory mediators worsen brain inflammation and act as neuromodulators, directly affecting neuron functions [86].

**Fig. 5:**
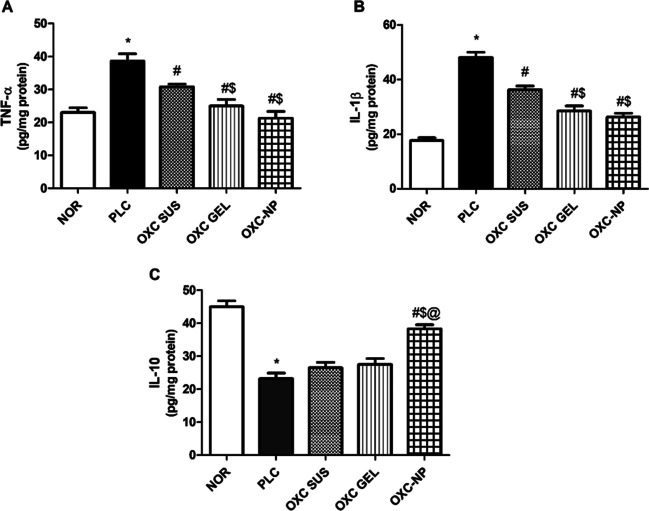
Effect of oxcarbazepine preparations on Hippocampal neuroinflammatory markers: (**A**) TNF-α, (**B**) IL-1β, (**C**) IL-10. Statistical analysis was done using one-way ANOVA followed by Student-Newman-Keuls multiple comparison test NOR = normal, PLC; group treated with pilocarpine; OXC SUS: group treated with oxcarbazepine suspension OXC GEL; group treated with oxcarbazepine in gel; OXC NP; group treated with oxcarbazepine chitosan nanoparticles in gel. # p<0.05 vs PLC, $ p<0.05 vs OXC SUS, @ p<0.05 vs OXC GEL.

Proinflammatory cytokines, namely, TNF-α and IL-1β, significantly influence epileptogenesis. Once released from microglia, they enhance glutamate transmission and neuronal hyper-excitability and activate a cascade of downstream inflammatory pathways [87]. In the current work, hippocampal TNF-α showed a significant increase (2-fold) in epileptic rats compared to the normal group. An effect previously reported with pilocarpine-induced seizures [88, 89]. Treatment with oxcarbazepine SUS, GEL, or OXC-NP induced a significant decline in the level of the inflammatory mediator (Fig. 5A). OXC GEL and OXC-NP showed the highest decrease in TNF-α compared with OXC SUS (p < 0.05). Comparable results were observed with IL-1β, where the PLC group showed a noticeable higher level of the inflammatory cytokine(p<0.05), as indicated in previous studies [88, 90]. OXC GEL and OXC-NP halted the rise in hippocampi IL-1β levels compared to PLC or OXC SUS (Fig. 5B). As recently reported, OXC suppressed hippocampal neuroinflammation via inhibiting microglial activation, thus reducing proinflammatory cytokines formation [91]. This effect was more pronounced with OXC-NP, indicating its preferential accumulation in the brain.

IL-10 represents a significant anti-inflammatory mediator that counterbalances inflammatory, insulting cytokines. In the brain, IL-10 suppresses inflammatory and apoptotic processes promoting neuronal survival and neurogenesis [92]. In this study, hippocampal levels of IL-10 were significantly decreased in epileptic rats. It was previously shown that temporal lobe epilepsy is accompanied by reduced IL-10 levels in animals [93], and humans [94]. OXC-NP was the only treatment showing restored IL-10 content (p < 0.05), whereas OXC SUS and OXC GEL failed to induce any change in its level compared to PLC (Fig. 5C). IL-10 was found to halt cytokine production, inflammasome activation, and immune response in an epileptic mouse model [95]. Such a finding contributed to the superior therapeutic potential of the OXC nanoformulation prepared in the current work.

**Fig. 6:**
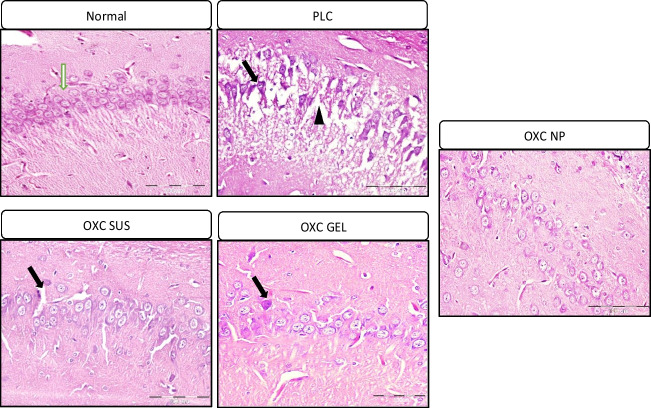
Photomicrographs of rats’ hippocampi (H&E, x400) treated with oxcarbazepine preparations. PLC; group treated with pilocarpine; OXC SUS: group treated with oxcarbazepine suspension OXC GEL; group treated with oxcarbazepine in gel; OXC NP; group treated with oxcarbazepine chitosan nanoparticles in gel. White head arrow = neuropil, black arrow = deeply stained cytoplasm, black head= neuronal disorganization.

Histopathological findings were parallel to previous findings. Control rats showed normal hippocampal tissue with well-organized, closely packed neurons and normally structured neuropil. Neurons have rounded cell bodies, basophilic cytoplasm, and vesicular nuclei. In the PLC group, extensive vacuolization of the neuropil, denoting axonal edema, was observed. In addition, diffuse neuronal injury occurs where neurons exhibit severe disorganization and appear shrunken with deeply stained cytoplasm (Fig. 6). Treatment with oxcarbazepine preparations exhibited different pathological profiles. A slight improvement was observed in the OXC-SUS group, which showed disorganized neurons and lost fibers in the neuropil area.

On the other hand, OXC GEL showed less neuronal disorganization with minimal vacuolization, but dark neurons were still observed. Treatment with OXC-NP revealed the best histopathological structure, with restored neuropil and well-organized neurons. Marginal and bi-nucleolus cells were found, indicating that neurons regained their activity (Fig. 6). Such findings support that OXC-NP possesses superior neuroprotection potential compared with OXC preparations.

### Correlations Investigated

**Fig. 7:**
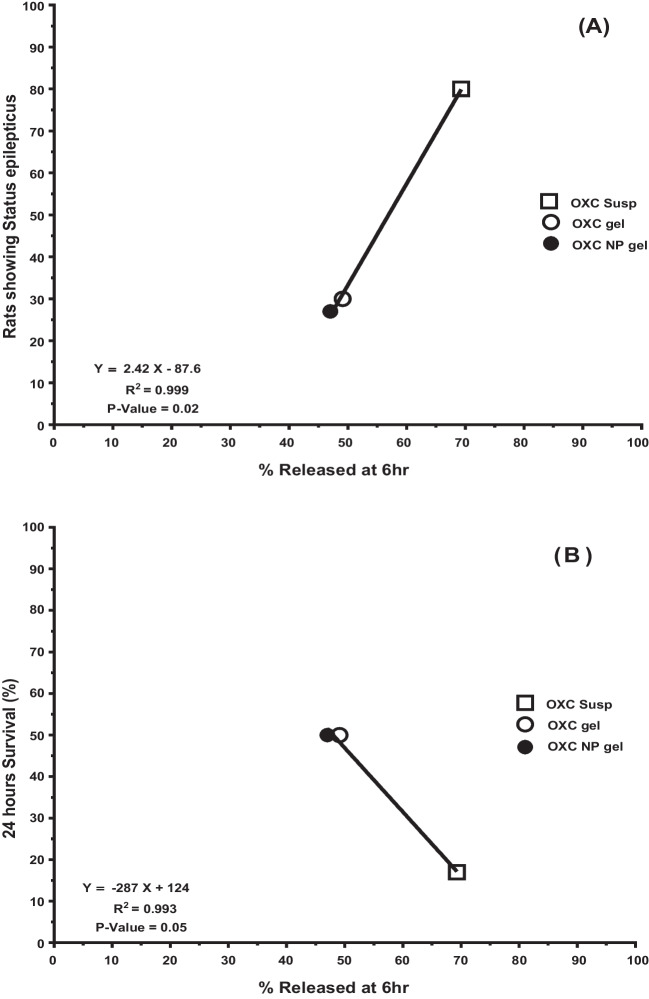
Correlation between % OXC released at 6 hrs and (**A**): Rats showing statues epilepticus, (**B**): 24 hours survival % of rats.

The quantitative correlation between *in- vivo* parameters and *in- vitro* pharmaceutical data was examined. The percent% drug-release at 6 h and the percent status of epilepticus rats revealed a significant correlation coefficient R^2^ = 0.999 (p = 0.02). In addition, the percentage drug release at 6 h showed a correlation coefficient value of R^2^ 0.993 with p-value = 0.05 when correlated with the percent 24 h survival (Table V) (Fig. 7).

**Table V:** Linear regression analysis of *in-vivo* behavior data in relation to percent Oxcarbazepine released at 6 hours for OXC suspension and both OXC loaded *in situ* gel and OXC NP-loaded *in situ* gel

Test	Linear regression (r^2^)	*P-*value
Latency to first seizure (sec ± SEM)	0.724	0.35
Average seizure score (score /group ±SEM)	0.940	0.16
Rats showing Status epilepticus (%)	0.999*	0.02*
24-hours survival (%)	0.993*	0.05*

*: The best correlation with significant p-value ≤ 0.05

## Conclusion

Administration of chitosan NP-loaded *in- situ* gel improves the delivery of OXC to the brain and enhances the pharmaceutical characteristics of the formulation. Furthermore, it was shown that chitosan NPs could carry drugs from the nose to the brain, and release them with improved residence times, and prolonged drug-release. This was manifested by the reduction in rats’ convulsive behavior induced by OXC-NP , as evidenced by the latency to the first seizure and seizure score compared to the free drug. In addition, rats showing status epilepticus and 24 h survival (%) data were significantly correlated with the percentage of drug released at 6 h. These findings suggest that intranasal mucoadhesive OXC-NP could represent a promising therapeutic system to effectively deliver oxcarbazepine to the brain in a low dose and thus with minimal systemic side effects.

## Supplementary Information

ESM 1 (DOCX 4895 kb)

## Data Availability

Data will be made available on request.
