# Supplementary material for: Thermoresponsive Gel-loaded Oxcarbazepine Nanosystems for Nose- To-Brain Delivery: Enhanced Antiepileptic Activity in Rats
Source: Pharm Res. 2023 Jun 23;40(7):1835–52. doi: 10.1007/s11095-023-03552-7 (PMC10421799; doi:10.1007/s11095-023-03552-7)
Supplement: Supplementary file 1 — (DOCX 4895 kb) [file 11095_2023_3552_MOESM1_ESM.docx]

**Supplementary Methods**

1. **TEM sample preparation procedure:**

Morphological examination of the optimized OXC CS- NP was done by transmission electron microscopy (TEM JSM-JEOL, Tokyo, Japan). OXC CS- NP fresh formulation was removed, cleaned and fixed by immersing them immediately in 4F1G (4 % formaldehyde: 1% glutaraldehyde) in phosphate buffer solution (PH= 7.2) at 4◦ C for 3 hours. Specimens were then post fixed in 2% OsO_4_ (OsO4 (osmium tetroxide) in the same buffer at 4◦C for 2 hours. Sample was washed in the buffer and dehydrated at 4◦ C through a graded series of acetone, then embedded in resin to polymerize. After that, sample was cut into sections about 90 angstrom in thickness. Place sections on grid cobber and staining by uranyl acetate for 5 min، then lead citrate for 2 min. After that, sample was examined under the transmission electron microscope and photos were taken.

1. **SEM sample preparation procedure:**

Morphological examination of the optimized OXC CS- NP was done by scanning electron microscopy (SEM JSM-JEOL, Tokyo, Japan). Small pieces of fresh specimens of OXC CS- NP were removed, cleaned and primary fixed by immersing them immediately in 4F1G aldehydes (4 % formaldehyde: 1 % glutaraldehyde) fixative in phosphate buffer solution (PH=7.4) at 4 °C for 3 hours. Specimens were then postfixed (secondary fixation) in 2% OsO_4_ (osmium tetroxide) in the same buffer at 4°C for 2 hours. Sample was washed in the buffer and dehydrated at 4°C through a graded series of ethanol. Sample of OXC CS-NP was dried, mounted using carbon paste on an AL- stub and coated with gold (conductive material) up to a thickness of 400 A in a sputter – coating unit (JFC-1100 E). Observations of OXC CS- NP morphology in the coded specimens were performed in a Jeol JSM-IT200 InTouchScope™ scanning electron microscope operated between 15 and 20 KeV.

**Fig. 1:** Optimized desirability plot (F4): Highest desirability value of F4 formula which is the optimized OXC-CS NP formulae with minimal PS, maximal EE, ZP and most appropriate PDI. (The highest desirability value in the case of optimized NP formulae was 0.999 for F4 formulae (with Chitosan concentration = 2 mg/ml & TPP concentration = 0.5 mg/ml))

**F4**

**Generalized seizure (score 5)**


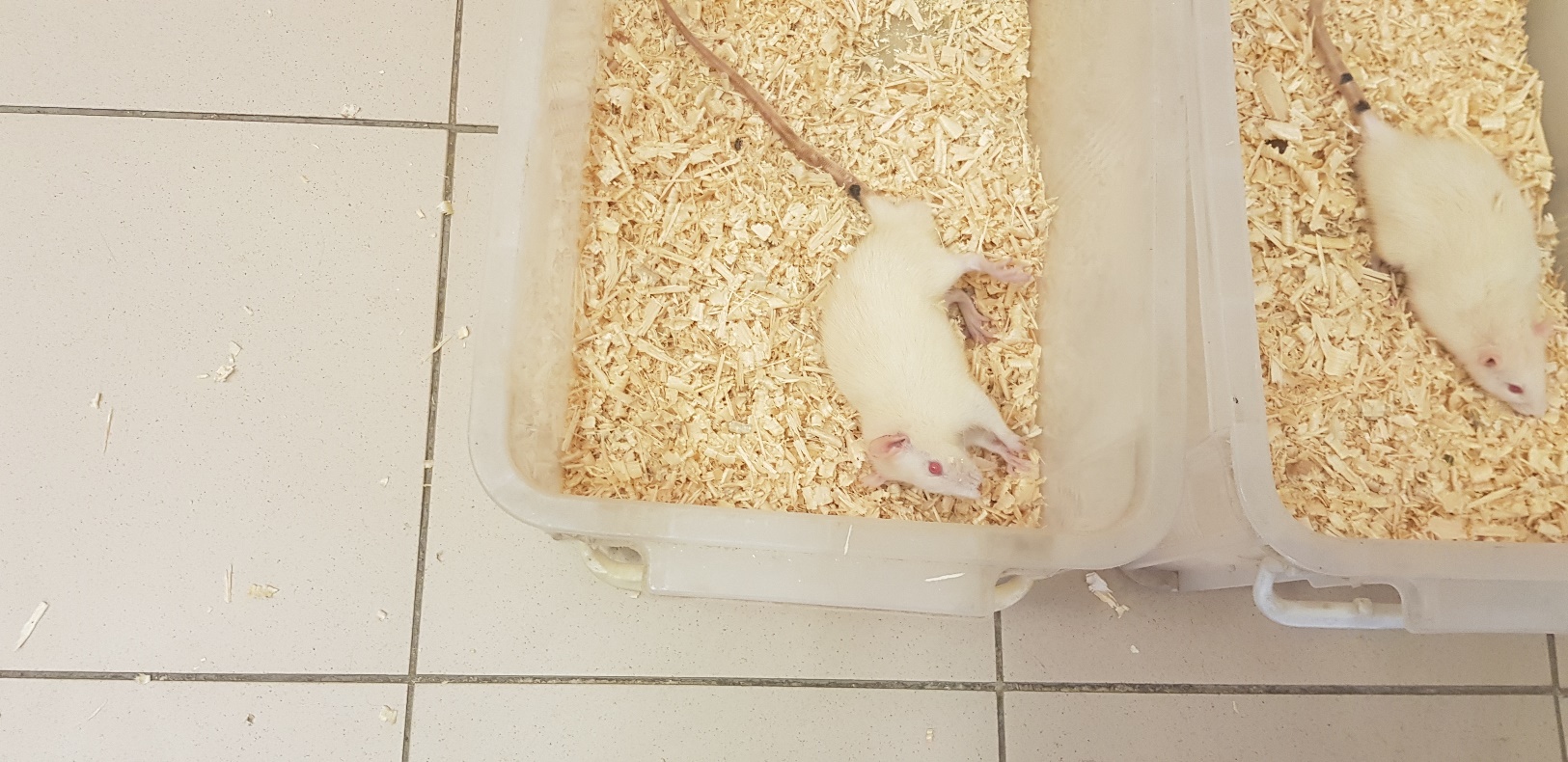

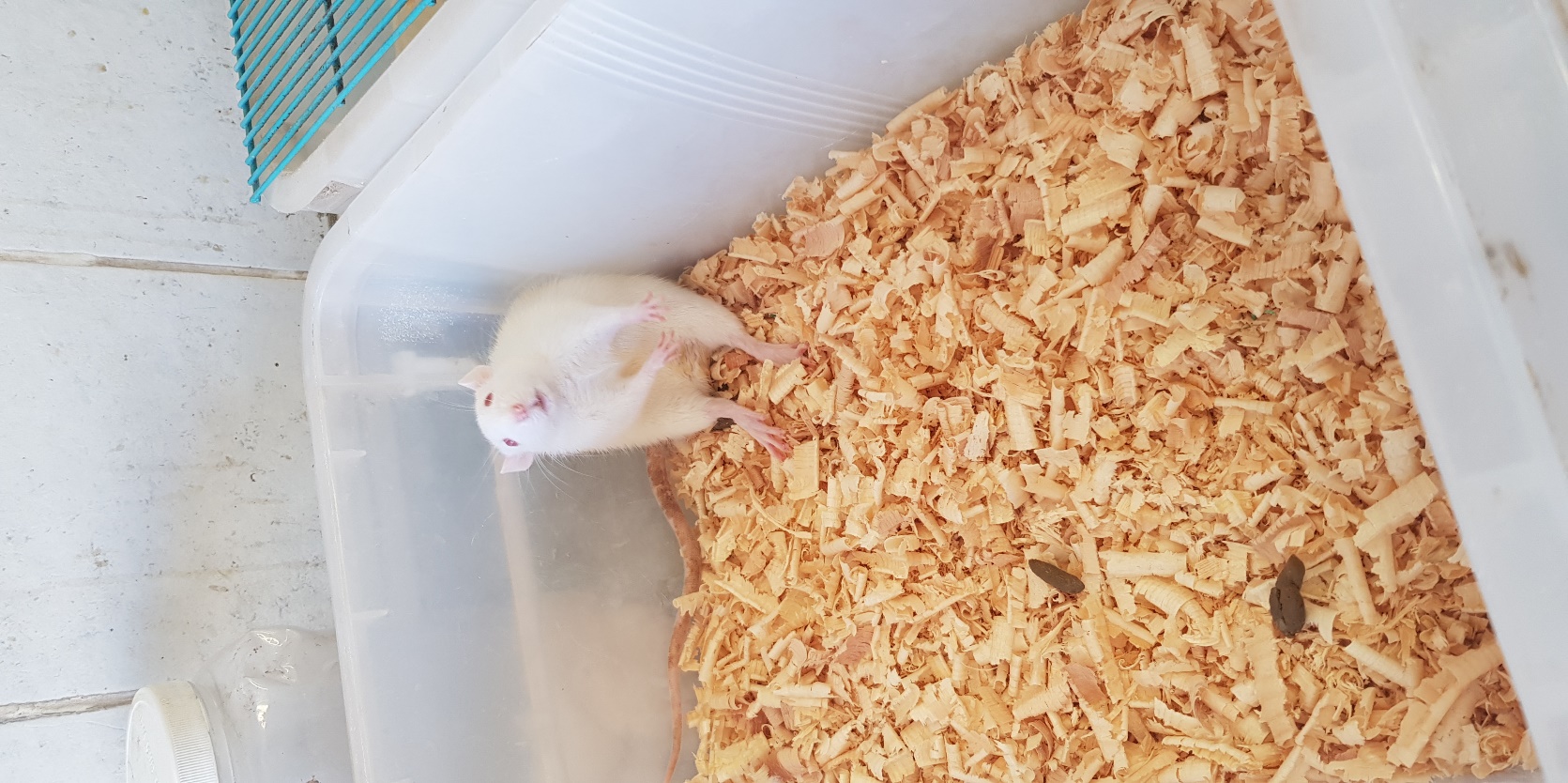


**Jumping and falling (score 5)**


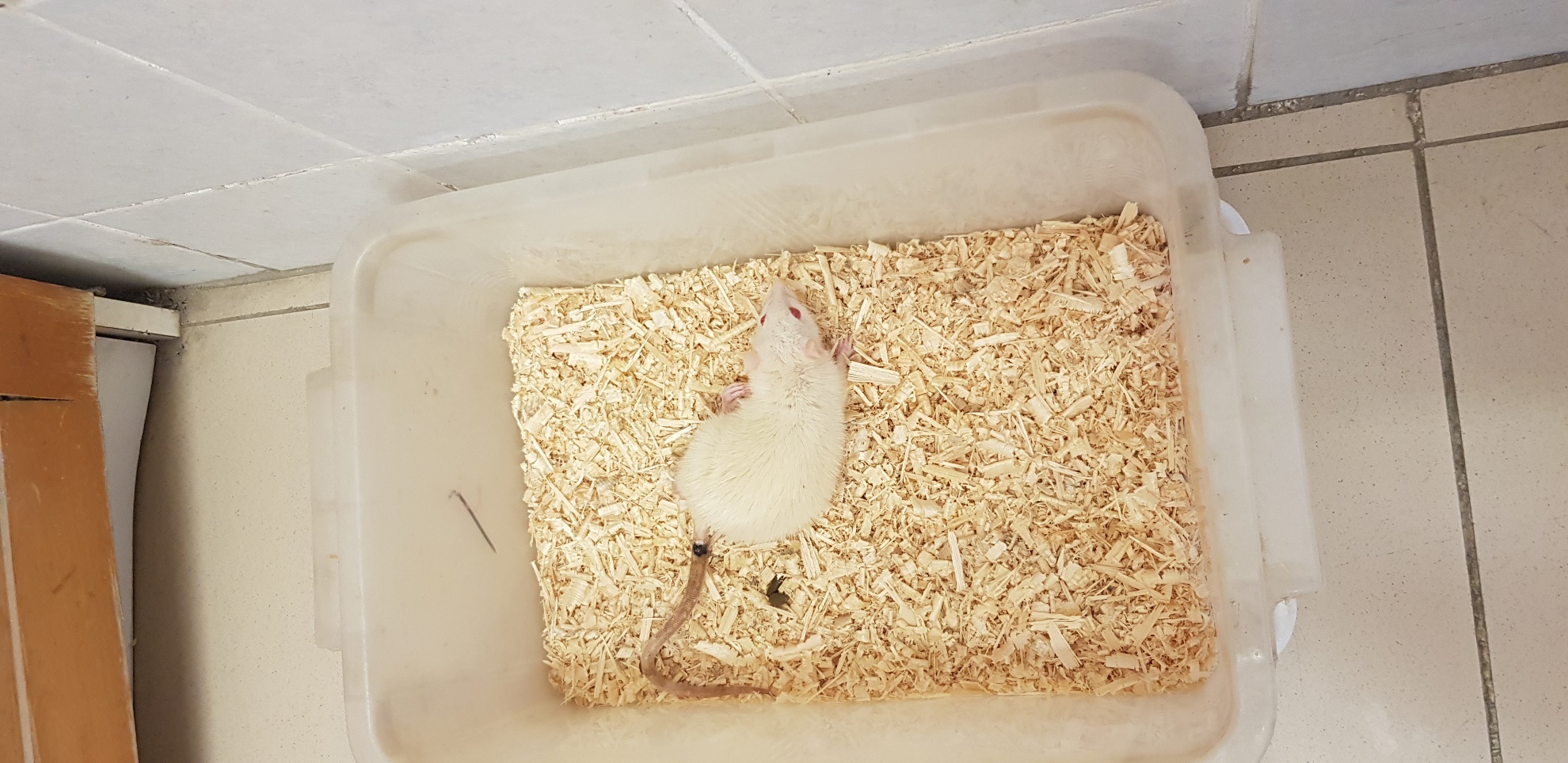


**Unilateral clonus (score 3)**

**Fig.2:** Representative photos showing seizure score of rats evaluated according to Modified Racine’s score
